# Management of Oral Anticoagulation and Antiplatelet Therapy in Post-Myocardial Infarction Patients with Acute Ischemic Stroke with and without Atrial Fibrillation

**DOI:** 10.3390/jcm11133894

**Published:** 2022-07-04

**Authors:** Francesca Romana Pezzella, Marilena Mangiardi, Mario Ferrante, Sebastiano Fabiano, Sabrina Anticoli, Fabrizio Giorgio Pennacchi, Antonella Urso, Leonardo De Luca, Valeria Caso

**Affiliations:** 1 Stroke Unit, Department of Neuroscience, San Camillo-Forlanini Hospital, 00152 Rome, Italy; sabrina.anticoli@gmail.com; 2 Unit of Neurology, Neurophysiology, Neurobiology, Department of Medicine, University Campus Bio-Medico, 00128 Rome, Italy; mario.ferrante1991@gmail.com; 3 Neuroradiology and Interventional Neuroradiology Department, San Camillo-Forlanini Hospital, 00152 Rome, Italy; sebastianofabiano@gmail.com; 4 Association for the Fight against Stroke (A.L.I.Ce. Lazio), 00179 Rome, Italy; fabrizio.pennacchi@gmail.com; 5 Health Department of Lazio Region, 00145 Rome, Italy; aurso-cons@regione.lazio.it; 6 Division of Cardiology, Department of Cardiosciences, San Camillo-Forlanini Hospital, 00152 Rome, Italy; ldeluca@scamilloforlanini.rm.it; 7 Stroke Unit, Santa Maria Della Misericordia Hospital, University of Perugia, 06156 Perugia, Italy; vcaso@hotmail.com

**Keywords:** acute ischemic stroke, atrial fibrillation, myocardial infarction, oral anticoagulation, antiplatelet therapy

## Abstract

The association between atrial fibrillation (AF), acute coronary syndrome (ACS), and stroke is a complex scenario in which the assessment of both thrombotic and hemorrhagic risk is necessary for scheduling an individually tailored therapeutic plan. Recent clinical trials investigating new antithrombotic drugs and dual and triple pathways in high-risk cardiovascular patients have revealed a new therapeutic scenario. In this paper, we review the burden of ischemic stroke (IS) in patients post-myocardial infarction with and without atrial fibrillation and the possible therapeutic strategies from a stroke point of view.

## 1. Introduction

After an acute myocardial infarction (MI), especially in the days immediately following, there is an increased risk of stroke, particularly ischemic stroke (IS) [1]. Several risk factors associated with post-MI acute stroke have been identified, including older age, female sex, prior IS, prior diabetes mellitus, atrial fibrillation, chronic kidney disease, heart failure during hospitalization, and ST-elevation myocardial infarction (STEMI) [2]. IS affects 0.9% of patients with MI within 1 month and 3.7% within 1 year after acute MI [3]; the 1-year mortality rate in these IS patients is double that for those not affected by stroke [4]. Although the mechanisms behind post-MI IS are largely unknown, the increased risk of IS after MI seems to be associated with pro-thrombotic factors, such as platelet activation, inflammation, and sympathetic activation [1].

Cardiovascular risk increases more after MI and in patients with established coronary artery disease if they develop AF. The incidence of AF after ACS ranges from 4 to 19%. Atrial fibrillation (AF) is the most common sustained arrhythmia, associated with a five-fold increased risk of acute ischemic stroke [4]. Furthermore, AF is an independent risk factor for ischemic stroke, and approximately one-third of patients with ischemic stroke have been found to have either clinical or subclinical AF [5], with a high prevalence of left atrial thrombosis. In the Framingham study, the age-adjusted incidence of IS was five-fold higher in subjects with AF, and the attributable risk increased from 1.5% at 50 to 59 years to 23.5% at 80 to 89 years [6].

### The Burden of Ischemic Stroke in Patients with and without Atrial Fibrillation Post-Myocardial Infarction

The coexistence of these two conditions may increase the risk of future cardiovascular events. In a non-selected cohort of 3960 individuals from the general population included in the ECHOES study, subjects with a prior history of myocardial infarct had a higher prevalence of AF, at around 6%, nearly three-fold higher than that of the general population [7]. Atrial fibrillation was also reported to significantly worsen post-MI patient outcomes and quality of life by increasing the risk of ischemic stroke up to 35-fold during follow-up [8]. A retrospective analysis conducted on the Danish National Patient Registry, with a total of 89,703 patients with MI analyzed with a 5-year follow-up, demonstrated that AF complicating MI early afterwards was an independent predictor for fatal or non-fatal stroke (HR: 2.34, 95% CI: 2.12–2.57, and HR: 2.47, 95% CI: 2.24–2.73, respectively) [9].

Additionally, Aronow (1989) [10] found that prior MI was a predictor of ischemic stroke in an elderly population (OR 4.84—*p* > 0.01); Ekowitz [11] observed that both MI and active angina pectoris increased the risk of ischemic stroke by 1.4 and 1.7, respectively. Gender differences were observed by Frost [12], who studied a random sample of the Danish population aged 50 to 89 years, with a hospital diagnosis of atrial fibrillation and no prior diagnosis of stroke. In this group, prior MI was a risk factor for stroke (both ischemic and hemorrhage) in men and not in women.

A six-study analysis noted that the incidence of stroke observed in patients with AF and a history of MI or angina pectoris was 5.6 per 100 person-years, in contrast to a rate of 1.4 in patients with AF alone [13].

As a consequence, current national and international guidelines consider MI a significant ischemic stroke risk factor in AF patients [14]. This condition of high ischemic cerebrovascular risk is certainly not negligible because, as previously discussed, coronary heart disease and myocardial infarction confer an increased risk of ischemic stroke in AF patients, and this condition coexists in 20% to 30% of these patients [15].

The REACH (Reduction of Atherothrombosis for Continued Health) registry of atherothrombosis enrolled a cohort of more than 26,000 patients with CAD; approximately 17% of them (N = 4460) had a history of cerebrovascular disease (stroke or TIA). Results from the 1- and 4-year REACH analyses showed that the incidence of major adverse cardiovascular events (MACE) increased over time, with the proportion of patients experiencing MACE being over 14% at 4-year follow-up [16].

In the GRACE registry [17] of acute coronary syndromes (ACSs), patients with a history of stroke represented 8.25% of the overall population.

Unsurprisingly, there is a growing interest in how to manage ischemic stroke in patients post-MI both with and without atrial fibrillation and to define the safest antithrombotic regimen to balance the bleeding and thrombotic risks.

## 2. Management of Antithrombotic Therapy in Post-Myocardial Infarction Patients with Stroke with and without Atrial Fibrillation: Lessons from Clinical Trials

Although there is a strong growing body of evidence for the management of patients with pre-existing AF presenting with acute MI [18], data on stroke prevention after acute MI are more limited, particularly for the patient without AF [19].

Patients with ACS and previous stroke/TIA have a higher risk of ischemic events, therefore, safely empowering antithrombotic therapy is desirable in these populations in order to balance the bleeding and thrombotic risks. Recently, new antithrombotic drugs, during or after acute coronary syndrome (ACS), were tested in several clinical trials (Table 1) However, in most of them, the cerebrovascular bleeding risk exceeded the antithrombotic benefit.

### 2.1. Clinical Trials in Combined Antithrombotic Drugs in Coronary Artery Disease (CAD) Patients

The TRACER trial [20] evaluated the association between vorapaxar (PAR-1 antagonist) and dual antiplatelet therapy (DAPT) with aspirin and clopidogrel in patients with ACS. In the study population, the combination of vorapaxar with standard therapy did not significantly change the primary composite endpoint of cardiovascular death, myocardial infarction, stroke, recurrent ischemia with urgent re-hospitalization, or coronary revascularization, but led to an increased risk of major bleeding, including intracranial hemorrhage (ICH), mainly in patients with a history of stroke.

In a secondary prevention setting, patients with a history of MI, acute ischemic stroke (AIS), or peripheral arterial disease (PAD) were challenged with 2.5 mg of vorapaxar. The TRA 2P–TIMI 50 trial [21] was discontinued in patients with a history of stroke owing to the risk of intracranial hemorrhage. However, vorapaxar reduced the risk of cardiovascular death or ischemic events in patients with stable atherosclerosis who were receiving standard therapy (aspirin and/or clopidogrel) and is recommended in patients with MI and PAD, while previous stroke (TIA/AIS and ICH) is a contraindication. Similarly, in the TRITON-Thrombolysis in Myocardial Infarction (TIMI) 38 trial [22], prasugrel was shown to reduce the rate of ischemic events and significantly increased the risk of ICH in patients with previous stroke or TIA; hence, a history of cerebrovascular events contraindicates prasugrel therapy. The TRITON-Thrombolysis in Myocardial Infarction (TIMI) 38 trial compared prasugrel vs. clopidogrel in patients with ACS treated by percutaneous coronary intervention. A post-hoc analysis showed that prasugrel reduced the rate of ischemic events, but in patients with previous stroke or TIA, it increased the risk of major bleeding (HR: 0.81, 95% CI: 0.73 to 0.90; *p* < 0.001). Indeed, a history of stroke/TIA represents a contraindication to prasugrel treatment [23].

Ticagrerol has been extensively studied in the cardiovascular patient population. In the PLATO [24] trial, ticagrerol, compared to clopidogel, reduced the risk of the primary composite ischemic outcome (cardiovascular death, MI, or stroke) in patients with ACS without significantly increasing the risk of ICH in subjects with a history of non-hemorrhagic stroke. The SOCRATES and the THALES trials were conducted in patients with non-cardioembolic, non-severe acute ischemic stroke, or high-risk transient ischemic attack. In the first study, ticagrelor monotherapy was not superior to aspirin in reducing the rate of stroke, myocardial infarction, or death at 90 days; however, in patients (about 25% of the trial population) with ipsilateral atherosclerotic stenosis, ticagrelor was superior to aspirin (6.7% vs. 9.4%, HR: 0.68 (0.53–0.88) *p* = 0.003), whereas in those with no ipsilateral stenosis, this effect was not observed [25,26]. In the THALES trial, ticagrelor combined with aspirin was superior to aspirin alone in patients with TIA and minor stroke for the prevention of stroke or death (5.5% vs. 6.6%, HR: 0.83 (0.71–0.96), *p* = 0.015) [26]. In patients with ipsilateral atherosclerotic stenosis, the absolute event rate for stroke or death within 30 days was higher in patients on aspirin alone (10.9%) than in patients on ticagrelor added to aspirin (3.0%) compared to patients without ipsilateral stenosis (5.3% and 0.5%, respectively). This is concordant with prior studies suggesting that atherosclerotic disease carries a greater risk than other stroke subtypes without stenosis among patients with TIA or minor ischemic stroke events on aspirin. Based on the results of the SOCRATES and THALES studies, treating patients with atherosclerotic stenosis with combination therapy, ticagrelor, and aspirin, could produce a clinically significant reduction in the relative and absolute risks of stroke and death compared to aspirin alone.

The rationale for combining anticoagulation and antiplatelet therapy for the acute treatment of ACS patients is well established. The rupture of atherosclerotic plaques is responsible for most acute coronary thrombosis events, and acute thrombus formation depends on both platelet aggregation and the coagulation cascade [27]. Accordingly, the current guidelines for the management of ACS recommend acute antiplatelet and anticoagulant therapy for hospitalized ACS patients regardless of whether or not percutaneous coronary intervention (PCI) is performed. As there is also evidence that thrombin generation persists for several months after ACS, combining oral antithrombin as part of a “dual pathway” can reduce the risk of recurrent thrombotic events and improve outcomes.

Several clinical trials in the 1990s—testing the association of ASA and warfarin—demonstrated lower odds of death, MI, or stroke compared with those observed with aspirin alone, but with a significant increase in major bleeding. Therefore, anticoagulation was never integrated into the standard of care for post-ACS patients who have no other indication for chronic anticoagulation.

In recent years, the APPRAISE-2 (Apixaban for Prevention of Acute Ischemic Events 2) [28] and the ATLAS ACS 2-TIMI 51 (Anti Xa Therapy to Lower Cardiovascular Events in Addition to ASA with or without Thienopyridine Therapy in Subjects with Acute Coronary Syndrome—Thrombolysis in Myocardial Infarction) [29] phase III clinical trials investigated the use of direct oral anticoagulants (apixaban and rivaroxaban, respectively) in the post-acute treatment of ACS. Both trials demonstrated a significant increase in major bleeding with their respective factor Xa inhibitors compared with dual antiplatelet therapy. In the latter study, in patients with previous stroke, apixaban was associated with worse outcomes regarding the primary efficacy endpoint (*p* for interaction = 0.08).

More encouraging results were shown by the COMPASS trial (Cardiovascular Outcomes for People Using Anticoagulation Strategies) [30], which investigated the use of rivaroxaban plus aspirin vs. aspirin monotherapy among patients with stable atherosclerotic vascular disease, 91% of whom had stable coronary artery disease. The composite primary outcome of cardiovascular death, stroke, or MI occurred less often among patients randomly assigned to 2.5 mg of rivaroxaban twice daily plus aspirin than among patients treated with aspirin alone (83 (0.9% per year) vs. 142 (1.6% per year); hazard ratio (HR), 0.58; 95% CI, 0.44–0.76). Ischemic/uncertain strokes were reduced by nearly half by the combination in comparison with aspirin alone (68 (0.7% per year) vs. 132 (1.4% per year); HR, 0.51; 95% CI, 0.38–0.68; *p* < 0.0001). No significant difference in the occurrence of stroke in the rivaroxaban-alone group in comparison with that for aspirin was noted (HR, 0.82; 95% CI, 0.65–1.05). Although major bleeding was higher in the rivaroxaban-plus-aspirin group (27 vs. 10; HR, 2.70; 95% CI, 1.31–5.58; *p* = 0.005), there was no increase in major/fatal bleeding or ICH (HR, 1.06; 95% CI, 0.72–1.56; *p* = 0.76; interaction *p* = 0.19).

The results of the COMPASS trial raise the possibility that long-term dual pathway inhibition by low-dose anticoagulation combined with low-dose antiplatelet therapy may be acceptably safe and substantially more effective than single pathway inhibition by antiplatelet therapy alone in preventing recurrent vascular events among patients with a history of atherosclerotic TIA and ischemic stroke.

From a stroke risk point of view, fatal and intracranial hemorrhage risk appear to be increased when a third antiplatelet medication (e.g., P2Y12 inhibitor) is included. The anticoagulant drug and dosage selection are also crucial: full-dose anticoagulation in the APPRAISE-2 study (apixaban at 5 mg twice daily) was associated with higher rates of major bleeding. However, very low doses of rivaroxaban (2.5 mg twice daily) were, overall, safe and efficacious in the COMPASS study.

As mentioned above, it is estimated that atrial fibrillation develops in up to 20% of patients with ACS, and these patients have higher stroke rates and in-hospital mortality than patients without atrial fibrillation. Their secondary prevention strategy is a complex clinical management issue that involves balancing thrombotic and bleeding risks.

### 2.2. Clinical Trials in Combined Antithrombotic Drugs in Atrial Fibrillation (AF) and Coronary Artery Disease (CAD)

A number of randomized controlled trials in acute MI patients with AF have assessed the effects of direct oral anticoagulants as an add-on therapy to dual antiplatelet therapy with aspirin and clopidogrel [31]; Table 2 summarizes the most recent studies.

The WOEST (What Is the Optimal Antiplatelet and Anticoagulant Therapy in Patients with Oral Anticoagulation and Coronary Stenting) trial compared DAPT and warfarin with clopidogrel (at a dose of 75 mg a day) and warfarin [32]. Without aspirin, fewer bleeding complications were noted.

The PIONEER AF-PCI (Open-Label, Randomized, Controlled, Multicenter Study Exploring Two Treatment Strategies of Rivaroxaban and a Dose-Adjusted Oral Vitamin K Antagonist Treatment Strategy in Subjects with Atrial Fibrillation Who Undergo Percutaneous Coronary Intervention) trial, in which patients were randomly assigned to one of two rivaroxaban strategies (low-dose rivaroxaban plus a P2Y12 inhibitor or very-low-dose rivaroxaban plus a P2Y12 inhibitor and low-dose aspirin) or triple therapy with warfarin, showed a lower rate of bleeding with each of the rivaroxaban treatment strategies than with triple therapy [33]. Moreover, the RE-DUAL PCI (Randomized Evaluation of Dual Antithrombotic Therapy with Dabigatran versus Triple Therapy with Warfarin in Patients with Nonvalvular Atrial Fibrillation Undergoing Percutaneous Coronary Intervention) trial randomly assigned patients to receive dabigatran with a P2Y12 inhibitor or warfarin-based triple therapy [34].

The AUGUSTUS (Antithrombotic Therapy after Acute Coronary Syndrome or PCI in Atrial Fibrillation) trial evaluated the independent effects of the oral anticoagulant apixaban and aspirin in patients with atrial fibrillation and a recent acute coronary syndrome or PCI (within the previous 14 days) [35]. Apixaban resulted in a lower bleeding rate than warfarin, and aspirin led to a higher bleeding rate than the placebo. The ENTRUST-AF PCI (Edoxaban Treatment Versus Vitamin K Antagonist in Patients with Atrial Fibrillation Undergoing Percutaneous Coronary Intervention) trial provided support for another DOAC as an option for patients with atrial fibrillation requiring antiplatelet therapy after PCI [36].

The above-mentioned studies demonstrated that it is safe to treat patients with an increased risk for bleeding with anticoagulation (warfarin studied in WOEST, rivaroxaban studied in PIONEER AF-PCI, dabigatran in RE-DUAL PCI, and edoxaban in ENTRUST-AF PCI) and P2Y12-inhibitor monotherapy. For patients with AF undergoing PCI, evidence suggests a regimen of DOACs plus a P2Y12 inhibitor was associated with fewer bleeding complications, including intracranial bleeding, without a significant difference in ischemic events compared with VKA plus DAPT. From a stroke point of view, it is important to highlight that all these studies have cerebrovascular events among the exclusion criteria; in this sense, the most restrictive was the PIONEER AF PCI, excluding all patients with a history of TIA or stroke, whereas the less restrictive was the RE-DUAL PCI trial, with stroke within 1 month among the exclusion criteria. Both the WOEST and ENTRUST-AF PCI trials did not enroll subjects with a history of ICH, and intracerebral vascular abnormalities are considered to be a significant risk for major bleeding.

## 3. Discussion

Finding the right balance between the prevention of stroke and recurrent coronary ischemic events compared to the risk of iatrogenic bleeding in patients with AF and SCA or elective PCI is challenging. 

A patient-centered approach carefully assessing the thrombotic and bleeding risks is required for each patient as part of a tailored treatment plan: the CHA2DS2-VASc score is the currently recommended risk stratification model for stroke prediction in AF; the HAS-BLED scale is one of the main tools for assessing the risk of major bleeding and is included in the latest ESC Guidelines for the diagnosis and management of atrial fibrillation developed in collaboration with the EACTS [37]. To mitigate the risk of ICH, an accurate bleeding evaluation is strongly recommended. The MICON risk scores, which include clinical variables and cerebral microbleeds, offer predictive value for the long-term risks of ICH and IS in patients prescribed antithrombotic therapy for secondary stroke prevention [38]. The assessment requires neuroimaging evaluation, which is currently part of standard stroke work. The S2TOP-BLEED score is another score that comprises ten variables (age, sex, smoking, modified Rankin Scale score, hypertension, diabetes, prior stroke, Asian ethnicity, BMI, and type of antiplatelet treatment). External validation of the score in a trial cohort and population-based cohort confirmed the robustness of the model [39]. 

Following this accumulation of evidence demonstrating the lack of benefit in terms of ischemic prevention with a consistent increase in the risk of bleeding complications associated with pretreatment, the 2020 ESC NSTEMI guidelines have recommended against the routine administration of P2Y12 inhibitors in patients in whom coronary artery anatomy is not known and an early invasive management is planned. Extended dual antithrombotic or antiplatelet regimen may be considered in case of a high-risk of an ischemic event with no high risk of bleeding. Several regimens may then be considered based on the results of the Prevention of Cardiovascular events in Patients With Prior heart Attack Using Ticagrelor Compared to Placebo on a Background of Aspirin-Thrombolysis in Myocardial Infarction (PEGASUS-TIMI 54), the Cardiovascular Outcomes for People Using Anticoagulant Strategies (COMPASS) and the Dual Antiplatelet Therapy (DAPT) trials [40]. 

In a recent meta-analysis that analyzed the efficacy and safety of dual antiplatelet therapy with aspirin and clopidogrel in patients with non-cardioembolic IS or transient ischemic attack, it was shown that in the long term DAPT doubles the risk of major bleeding. However, the major bleeding risk did not further increase in older patients compared with younger patients, indicating a risk ceiling effect of ≈3% per year. This effect is probably due to the fact that patients with high bleeding risks were not included in the trials due to strict exclusion criteria or that the major bleeding events led to premature death [41,42].

Moreover, DOAC-based treatment strategies should be preferred over VKA in patients eligible for DOACs, and the duration of triple therapy should be minimized effectively to reduce bleeding risks.

For many patients, the DOAC-plus-P2Y12-inhibitor combination immediately after ACS and/or PCI would be optimal. For patients taking oral anticoagulants, in secondary long-term prevention, clopidogrel is recommended over other P2Y12 inhibitors (e.g., prasugrel and ticagrelor) in combination.

The timeline of antithrombotic therapy in AF and coronary artery disease must be tailored to patients at risk of thrombosis and bleeding. For patients with high thrombotic risk (including ACS), ≥3 months (and ≤12 months) of clopidogrel and ≤1 month of aspirin (ASA) is recommended.

Longer treatment courses for NOAC-associated clopidogrel may be appropriate for patients at a high ischemic risk or experiencing ACS. For PCI for stable angina, a shorter course of clopidogrel and ASA (≤7 days) may be more appropriate.

Despite the countless amount of data analyzed that come from clinical trials, it is difficult to draw univocal conclusions that can lead to the drafting of guidelines. This is probably due to the fact that the population examined in the trials is very heterogeneous, that patients at a high risk of bleeding are often excluded, and that the subgroups among the various populations do not always reach statistically significant numbers.

Finally, it is necessary to underline that in stroke patients the scenarios can be multiple based on the stroke site, extension of ischemic lesion and the related disability.

Based on these considerations, we can affirm that the clinical trials are useful to make us aware of the best pharmacological treatment to be used in patients suffering from ischemic stroke and myocardial infarction, associated or not with AF, without, however, losing sight of clinical practice by balancing the benefit–risk ratio for each patient.

## 4. Conclusions

The optimal strategy for managing complex and high-risk cardiovascular patients requires an individually tailored approach to realize the maximum benefit and minimize ICH and bleeding risk. Registries and data from observational studies are critical for understanding how trial results are implemented in the real world and what effects are yielded. Recently, the ***Cardiology Council on Stroke in cooperation with the European Stroke Organisation*** conveyed a two-day workshop to discuss current and emerging concepts and apparent gaps in stroke care, including risk factor management, acute diagnostics, treatments and complications, and operational/logistic issues for health care systems and integrated networks. Joint initiatives of cardiologists and stroke physicians are needed in research and clinical care to target unresolved interdisciplinary problems and to promote the best possible outcomes for patients with stroke [43].

In this review, we focused on providing an overview based on the many complex clinical trials that have examined the balance between the efficacy and related risks of antiplatelet and anticoagulant therapies, alone or combined, in patients affected by myocardial infarction and/or stroke.

Furthermore, this review might be useful for clinicians, cardiologists, and neurologists, ready to apply the best therapeutic algorithm in accordance with managing the bleeding risk in patients.

## Figures and Tables

**Table 1:** Trials of combined antithrombotic drugs in coronary artery disease (CAD) patients.

Trial Name	TRACER (2018)	TRA-2P (2012)	TRITON-TIMI (2009)	PLATO (2009)	SOCRATES (2017)	APPRAISE-2 (2015)	ATLAS (2011)	COMPASS (2019)
Patient Population	12,944 ACS patients without ST elevation.	26,449 patients who had a history of MI, IS, or PAD.	13,608 patients with ACS.	18,624 ACS patients with or without ST elevation.	13,199 patients >40 years with a non-cardioembolic, non-severe acute IS, or high-risk of TIA.	7392 patients with recent ACS and additional risk factors for recurrent ischemic events.	15,526 patients with recent ACS.	27,395 patients with stable CAD or PAD.
Treatment	Vorapaxar 2.5 mg daily (PAR-1 antagonist) + DAPT vs. placebo.	Vorapaxar (PAR-1antagonist) 2.5 mg daily vs. placebo.	Prasugrel (thienopyridine) vs. clopidogrel.	Ticagrelor (reversible P2Y12 inhibitor) vs. clopidogrel.	Ticagrelor (reversible P2Y12 inhibitor) vs. aspirin.	First group Apixaban 5 mg twice daily. Second group Placebo All patients received DAPT.	First group Rivaroxaban 2.5 mg twice daily. Second group Rivaroxaban 5 mg twice daily. Third group Placebo All patients received DAPT.	First group Rivaroxaban 2.5 mg twice daily + aspirin 100 mg daily. Second group Rivaroxaban 5 mg twice daily. Third group Aspirin 100 mg daily.
Ischemic endpoint	Death from cardiovascular causes, MI, IS, recurrent ischemia with rehospitalization, or urgent coronary revascularization.	Death from cardiovascular causes, MI, or IS.	Death from cardiovascular causes, nonfatal MI, or nonfatal stroke.	Death from cardiovascular causes, MI, or IS.	Time to occurrence of IS, MI, or cardiovascular death within 90 days.	Death from cardiovascular causes, MI, or IS.	Death from cardiovascular causes, MI, or IS.	Death from cardiovascular causes, MI, or IS.
Bleeding endpoint	Moderate or severe bleeding (according to GUSTO * classification).	Moderate or severe bleeding (according to GUSTO * classification).	TIMI major bleeding not related to CABG.	Major bleeding.	Life-threatening bleeding or major or minor bleeding.	TIMI major bleeding.	TIMI major bleeding not related to CABG.	Modified ISTH major bleeding.
Ischemic events	No significant reduction (HR 0.92; *p* = 0.07).	Reduction (HR 0.87; *p* < 0.001).	Reduction (HR 0.81; *p* < 0.001).	Reduction (HR 0.84; *p* = 0.001).	6.7% of patients with ipsilateral stenosis in ticagrelor group 9.6% in aspirin group.	No significant reduction (HR 0.95; *p* = 0.51).	Reduction (HR 0.84; *p* = 0.008); Rivaroxaban 2.5 mg, 1.8%; Rivaroxaban 5 mg, 2.4%; Placebo, 0.6%; Rivaroxaban 2.5 mg vs. placebo, HR 3.46 (95% CI, 2.08–5.77); Rivaroxaban 5 mg vs. placebo, HR 4.47 (95% CI, 2.71–7.36).	Rivaroxaban + aspirin vs. aspirin, HR 0.76 (95% CI, 0.66–0.86). Rivaroxaban vs. aspirin, HR 0.90 0 (95% CI, 0.79–1.03).
Bleeding events	Increased (HR 1.35; *p* < 0.001); increased ICH 1.1% vs. 0.2% (*p* < 0.001).	Increased (HR 1.66; *p* < 0.001); ICH 1.0%, vs. 0.5% in the placebo group; (*p* < 0.001).	Increased (HR 1.32; *p* = 0.03).	No significant increase (*p* = 0.43).	Increased in ticagrelor group (HR 1.68 vs. 1.23; *p* = 0.09 vs. *p* = 0.22).	Increased risk of major bleeding (HR 2.59; *p* = 0.001).	Increased 2.1% vs. 0.6% (*p* < 0.001); ICH 0.6% vs. 0.2% (*p* = 0.009).	Rivaroxaban + aspirin vs. aspirin, HR 1.70. Rivaroxaban vs. aspirin, HR 1.51.
Bleeding events in previous stroke/TIA	Increased but no significant interaction (*p* = 0.771).	Increased 2.4%, as compared with 0.9% in the placebo group (*p* < 0.001).	Increased (HR 2.46; *p* = 0.22).	No significant increased (*p* = 0.77).	Increase in patients with lacunar stroke in ticagrelor group.	No significant increase (*p* = 0.31) but worse outcome regarding ischemic events.	Four events in Rivaroxaban group; none in placebo group (*p* not available).	Rivaroxaban plus aspirin = 2, rivaroxaban alone = 3, aspirin alone = 0 (*p* not available).

ACS: acute coronary syndrome; DAPT: dual antiplatelet therapy; MI: myocardial infarction; IS: ischemic stroke; PAD: peripheral arterial disease; TIMI: thrombolysis in myocardial infarction; CABG: coronary artery bypass graft; ISTH: International Society on Thrombosis and Hemostasis. * Global Utilization of Streptokinase and Tissue Plasminogen Activator for Occluded Coronary Arteries.

**Table 2:** Trials of combined antithrombotic drugs in atrial fibrillation (AF) and coronary artery disease (CAD).

Trial Name	WOEST (2009)	PIONEER AF-PCI (2015)	RE-DUAL PCI (2016)	AUGUSTUS (2020)	ENTRUST-AF PCI (2019)
Patient Population	573 total patients, 27% with ACS, taking OAC undergoing PCI.	2124 total patients, 51.6% with ACS, with AF, undergoing PCI.	2725 total patients, 64% with ACS, with AF, undergoing PCI.	4614 total patients, 60.9% with ACS, with AF and recent ACS or PCI.	1506 total patients, 51.6% with ACS, with AF and recent ACS or PCI.
Treatment	Group 1: OAC + clopidogrel (double therapy); Group 2: OAC + DAPT with clopidogrel (triple therapy).	- Group 1: 15 mg of rivaroxaban daily + clopidogrel; - Group 2: 2.5 mg of rivaroxaban twice daily + DAPT; - Group 3: VKA + DAPT.	Group 1: −110 mg of dabigatran twice daily + clopidogrel or ticagrelor; Group 2: 150 mg of dabigatran twice daily + clopidogrel or ticagrelor; Group 3: VKA + DAPT with clopidogrel or Ticagrelor.	Group 1: 5 mg of apixaban twice daily + DAPT; Group 2: 5 mg apixaban twice daily + P2Y12 only; Group 3: VKA + DAPT; Group 4: VKA + P2Y12 only.	Group 1: 30–60 mg of edoxaban daily + P2Y12; Group 2: VKA + DAPT.
Primary Outcome	Any bleeding episode within 1 year of PCI.	Thrombolysis in myocardial infarction; major bleeding; bleeding requiring medical attention, and minor bleeding.	Major or clinically relevant non-major bleeding event.	Major and clinically relevant non-major bleeding.	Major and clinically relevant non-major bleeding.
Bleeding Outcome Rate	Double therapy, 19.4%; Triple therapy, 44.4%; Double vs. triple, HR, 0.36 (95% CI, 0.26–0.50).	Group 1, 16.8%; Group 2, 18.0%; Group 3, 26.7%; 1 vs. 3, HR, 0.59 (95% CI, 0.47–0.76); 2 vs. 3, HR, 0.63 (95% CI, 0.50–0.80).	D110 + P2Y12, 15.4%; D150 + P2Y12, 20.2%; VKA + DAPT, 26.9%; D110 vs. TT, HR, 0.52 (95% CI, 0.42–0.63); D150 vs. TT, HR, 0.72 (95% CI, 0.58–0.88).	Apixaban, 10.5%; VKA, 14.7%; DAPT, 16.1%; P2Y12 only, 9.0%; Apixaban vs. VKA, HR, 0.69 (95% CI, 0.58–0.81); DAPT vs. P2Y12, HR, 1.89 (95% CI, 1.59–2.24).	Edoxaban, 17%; VKA, 20%; Edoxaban vs. VKA, HR, 0.83 (95% CI, 0.65–1.05).
Bleeding Events	Increased (HR, 1.35; *p* < 0.001); increased ICH, 1.1% vs. 0.2% (*p* < 0.001).	Increased (HR, 1.66; *p* < 0.001); ICH, 1.0%, vs. 0.5% in the placebo group; (*p* < 0.001).	Increased (HR, 1.32; *p* = 0.03).	No significant increased (*p* = 0.43).	Increased in ticagrelor group (HR, 1.68 vs. 1.23; *p* = 0.09 vs. *p* = 0.22).

ACS: acute coronary syndrome; DAPT: dual antiplatelet therapy; OAC: oral anticoagulant; TT: triple therapy; VKA: vitamin K antagonist; PCI: percutaneous coronary intervention.
